# Australian child and adolescent mental health services

**DOI:** 10.1192/bji.2024.4

**Published:** 2024-08

**Authors:** Paul Robertson, Valsamma Eapen

**Affiliations:** 1 Associate Professor, Department of Psychiatry, University of Melbourne, Melbourne, Australia; 2 Professor of Infant, Child and Adolescent Psychiatry, Discipline of Psychiatry and Mental Health, School of Clinical Medicine, University of New South Wales, Sydney, Australia. Email: v.eapen@unsw.edu.au

**Keywords:** Australia, children and adolescents, mental health, child and adolescent mental health services, service delivery

## Abstract

We aim to describe the Australian child and adolescent mental health system, which has its historical origins in the child guidance clinic, with recent efforts at modernisation to meet community needs and major policy innovations, including the National Disability Insurance Scheme (NDIS) and expansion of digital/telehealth services. Shared funding/responsibility across commonwealth and state/territory governments has resulted in country-wide variations, allowing innovation but also introducing fragmentation and duplication. The increase in demand outstripping supply (which was exacerbated by workforce shortages resulting from the pandemic), the lack of robust evaluation, and poor service integration (which make navigation difficult for families) are ongoing challenges.

The Commonwealth of Australia comprises six states and two territories. Australia has strong government and public service systems supporting health, education, employment and social services. While maintaining strong ties with the UK and USA, Australia has a close relationship with New Zealand and increasingly sees itself within the Asia Pacific region. The Australian child and adolescent mental health (CAMH) system is embedded in the broader mental health and health system.

Australia's population is around 25 million, with just over 20% aged under 18 years. Indigenous Australian history dates back over 50 000 years, with contemporary Australia born through British colonialisation over the past 250 years. Modern Australia is highly multicultural and over 85% live in coastal areas, leaving regional/rural areas sparsely populated. These demographic and geographical factors make delivery of effective and equitable healthcare a challenge.

Total health spending is 9.8% of gross domestic product, with 7.3% of the budget spent on mental health but only a fraction of this is allocated to CAMH. Both state and Commonwealth governments are active in CAMH policy, funding and service delivery, with a National Children's Mental Health and Wellbeing Strategy developed in 2021.^1^ The Commonwealth-funded health insurance (Medicare) covers primary care and community office-based specialist mental health practice on a ‘fee for service’ model, often with a patient co-payment, referred to as the ‘private sector’. This insurance includes psychiatric consultation and care and psychotherapeutic treatments by psychologists and other mental health clinicians. State-funded services cover hospital and specialist community CAMH services, while developmental/intellectual disability and mental health-related (categorised as psychosocial) disability are covered by the National Disability Insurance Scheme (NDIS) funded by the Commonwealth government. Further, non-governmental organisations (NGOs) deliver a range of CAMH-relevant services, such as youth, family and drug and alcohol, services, out-of-home care (foster or residential care) and employment support/recovery or rehabilitation. The Commonwealth also directly funds headspace, a network of youth-focused (12–25 years of age) mental health clinics.^2^ Head to Health Kids is a similar development for children 0–11 years old. This complex policy, governance and funding environment provides CAMH services with some national consistency in themes and architecture, but leads to substantial variation in service form, level and nomenclature across states and territories. Consequently, a universal description of the Australian CAMH system is challenging.

The epidemiology of child and adolescent (4–17 years) mental health difficulties in Australia is comparable to international estimates, with a prevalence of around 14%.^3^ Significant increase in demand and expansion of digital (e.g. parenting programmes) and telehealth service delivery that occurred during the pandemic has improved access particularly for those in regional/rural areas. Although well-resourced by international standards, there remains a substantial shortfall, with only a quarter of those with significant mental health problems seeing child psychiatrists, while the others access general practitioners (GPs), paediatricians and psychologists.^4^

Australia has recently seen considerable political will, additional funding and efforts to modernise its mental health system, including high-level inquiries^5^ outlining the argument and impetus for transforming the mental healthcare system, including CAMH services, but with variable response from states and territories. A tiered CAMH system is envisaged, with strong community orientation and an integrated continuum of connect and care (I-CCC) pathways to higher- or lower-intensity services from primary to specialised, as individual needs change.^6^

The vision for change also calls for a cultural change, with an emphasis on human rights and greater voice for those with lived experience and the emergence of a peer workforce. Much of this is articulated in adult mental health and the process of bringing young peoples’ voices into the design, running and delivery of CAMH services is in its infancy.

An aspect of Australian CAMH has been the emergence of the youth mental health movement,^2^ including the change from CAMH to CY(outh)MH services, covering 0–25 years of age. This represents Australia's response to transitional (transition-age) youth, and at the younger end of the age range, Australia has more developed perinatal and infant mental health services than elsewhere.

Despite some of the recent innovations in models of care and expanded funding, the CAMH service system is facing significant challenges in workforce size, architecture and equitable distribution of resources. This is further compounded by poor integration across state and commonwealth governments; across health, disability, education and social care within each government; and between government, ‘private’ and NGO sectors.

## Architecture of the Australian CAMH system

The aspiration is for a CAMH system that is community oriented, accessible and providing the right intensity of care, at the right time and close to home, with the capacity and flexibility to step up and down the care levels.

### Prevention, early intervention and primary care

Prevention and early intervention services are currently expanding and include the developmental surveillance programme and early parenting interventions. There is a renewed focus on the first 2000 days from pregnancy to the start of school,^7^ with headspace and youth mental health conceived as early (in illness) intervention.

Australia has a robust primary care sector, with GPs serving as the first point of contact and the referral conduit to more specialised CAMH care. Limitations in time, training and skills, funding models and access to psychiatric consultation due to cost and low availability are barriers to greater CAMH service delivery in primary care, although it is expanding.

### Middle-tier CAMH services

The middle-tier services such as headspace and, more recently, the Head to Health hubs, initially conceptualised as enhanced primary care, are increasingly providing care for those with moderate to severe mental disorders that do not meet the clinical or capacity thresholds of the tertiary public CAMH services; in other words, closing the ‘missing middle’. However, there is debate about headspace's clinical and cost-effectiveness, whether it targets those most in need and whether it is worsening the fragmentation and lack of integration within the service system.^8^

Medicare-funded, private office-based practice by psychiatrists, paediatricians and allied health clinicians has recently seen substantial investment by the Commonwealth, which has also increased access to subsidised psychotherapeutic treatments in the community through Medicare. However, the substantial ‘gap’ payments paid by the patient (a charge determined by the practitioner above the government rebate) and unequal distribution limits its access for regional/rural and disadvantaged populations with the greatest need.

### Recovery- or rehabilitation-focused intervention

The NGO sector, using state and Commonwealth funding, delivers recovery and rehabilitation intervention, including NDIS-funded interventions for those with a disability, drug and alcohol services and a range of CAMH-relevant therapeutic services (e.g. family support, child protection, out-of-home (foster or residential) care). However, poor integration and diffusion of accountability across public and private bodies and NGOs, and across levels of government, risk duplication and service inefficiencies while also making the system confusing and difficult for families to navigate – concerns well articulated in recent inquiries^5^ and the NDIS review.^9^

### Specialist CAMH services

The state and territory government-funded CAMH services provide multidisciplinary child and adolescent in-patient and out-patient care, intensive mobile outreach, acute and urgent crisis response, triage services, consultation to emergency departments, and specialised programmes for eating disorders, early psychosis, neurodevelopmental conditions etc., and provide consultation–liaison services when based in paediatric hospitals/centres. Historically, the Australian CAMH model was similar to that in the UK, providing ongoing intensive, specialised care, but recently it has shifted the focus to shorter interventions for severe, complex and crisis-driven urgent presentations. CAMH services typically cover less than 1% of under-18-year-olds. This coverage, and also access to adolescent mental health in-patient beds, is substantially less than in comparable countries.^10^ Australian CAMH relies on a robust community CAMH service, with in-patient care being an unusual intervention with varying availability across states. For example Victoria has 60 adolescent short-term in-patient beds for a population of 6–7 million, whereas Tasmania has no in-patient beds and only limited access to paediatric beds. Although step-down residential units jointly managed by NGOs and mental health services are emerging, including Youth Prevention and Recovery Care (YPARC) centres and some options for supported residential care,^11^ significant gaps remain a challenge.

## CAMH workforce

The Australian CAMH workforce consists of psychiatrists; paediatricians; allied health professions, including clinical psychologists, occupational therapists and social workers; and nurses. Child and adolescent psychiatry is a subspecialty of psychiatry requiring a 2-year training within a 5-year psychiatry fellowship training under the Royal Australian and New Zealand College of Psychiatrists (RANZCP).^12^ Paediatricians deliver more CAMH services than in comparable countries, including assessment and management of attention-deficit hyperactivity disorder, autism and emotional and behavioural problems. Allied health professionals provide case management and psychotherapeutic treatments. Considerable training occurs within state CAMH services, and the peer workforce, both patient and carer/parent, is gaining prominence.

Most specialist CAMH services and paediatric hospitals/centres have university affiliations and conjoint academic–clinical positions with a focus on research and teaching. Academic child and adolescent psychiatry has a strong tradition in Australia, although currently there are significant workforce concerns.

Australia boasts excellent workforce and training standards, but the current CAMH workforce shortage is a major challenge. Although there is a need to increase the workforce size and capacity, in the interim ensuring that specialist skills are deployed in the most effective way to those most in need will enable an accessible, effective, equitable and sustainable CAMH care system.

## Data Availability

Data availability is not applicable to this article as no new data were created or analysed in this study.
